# Health-related quality of life of HIV infected adults with and without Visceral Leishmaniasis in Northwest Ethiopia

**DOI:** 10.1186/s12955-017-0636-6

**Published:** 2017-08-30

**Authors:** Mekuriaw Alemayehu, Mamo Wubshet, Nebiyu Mesfin, Aschalew Tamiru, Abebaw Gebayehu

**Affiliations:** 1 Institute of Public Health, College of Medicine and Health Sciences, University of Gondar, P. O. Box - 196, Gondar, Ethiopia; 2 Department of Public Health, St. Paul’s Hospital Millennium Medical College, Addis Ababa, Ethiopia; 3 School of Medicine, College of Medicine and Health Sciences, University of Gondar, Gondar, Ethiopia; 4 Leishmaniasis Research and Treatment Center, College of Medicine and Health Sciences, University of Gondar, P. O. Box – 196, Gondar, Ethiopia

**Keywords:** Health-related quality of life, Human Immunodeficiency virus, Visceral Leishmaniasis, Coinfection

## Abstract

**Background:**

Health-related quality of life (HRQoL) is an important outcome measure among HIV infected patients receiving antiretroviral therapy (ART). When HIV infected patients coinfected with Visceral Leishmaniasis (VL) the problem become severe because VL accelerates HIV replication and disease progression. The impact of VL on the quality of life of HIV infected patients has not been studied. In this study in Ethiopia, we compared the quality of life of HIV infected patients with and without VL.

**Methods:**

A cross-sectional study was conducted from October 2015 to September 2016 in selected health centers and hospitals, in Northwest Ethiopia. Data on quality of life was collected by trained nurses. The instrument used to collect the data was the short Amharic version of the World Health Organization Quality of Life for HIV clients (WHOQoL-HIV). Depression was assessed using the validated version of Kessler scale. Data was entered and analyzed using SPSS version 20. Descriptive statistics, bivariate and multivariate linear regression model was used to summarize the results.

**Results:**

A total of 590 study participants were included in the study with response rate of 95%. Of the 590 patients included in our study 125 (21%) were HIV-VL coinfection. HIV-VL coinfected patients had a lower quality of life in all the domains as compared to HIV patients without VL. Depression was consistently and strongly associated with all the quality of life domains of both groups. Also, in HIV infected patients a longer duration in ART was associated with higher HRQoL domains except for the spiritual and level of independence domains. With regard to HIV-VL coinfected patients, a longer duration in ART was associated with psychological, spiritual and level of independence domains of HRQoL. Demographics, clinical, and treatment characteristics resulted few significant associations with HRQoL domains of both groups.

**Conclusion:**

HIV-VL coinfected patients had a poor quality of life in all the domains of the WHOQoL-HIV instrument. Depression, duration of ART and education were strongly associated with the quality of life. Depression should be targeted for intervention to improve the quality of life.

## Background

Visceral leishmaniasis (VL; also known as “kala-azar”) is a systemic parasitic disease caused by the parasite of *Leishmania donovani* species complex. It is estimated about 500,000 new cases of VL occur annually [1]. VL is characterized by irregular bouts of fever, substantial weight loss, swelling of the spleen and liver, and anemia (which may be serious). If the disease is not treated, the fatality rate in developing countries can be as high as 100% within 2 years [2]. VL accelerates HIV replication and disease progression, mainly by chronic immune stimulation [3].

In India and particularly in Africa, both HIV and VL infection (hereafter, “HIV-VL coinfection”) is emerging. The AIDS pandemic has expanded to rural areas where VL is endemic, with cases of HIV-VL coinfection reported in 35 countries [4, 5], among which Ethiopia carries the greatest burden. The prevalence of HIV and HIV-VL coinfection in Ethiopia is 1.1% [6] and 17.75% [7] respectively.

The advent of antiretroviral therapy (ART) and its widespread availability in many settings has reduced the mortality rate among people living with HIV/AIDS (PLHA) [8]. As longevity of PLHA improves as a result of ART, improvement of quality of life (QoL) of these patients has become an important issue for researchers and policy makers [9]. Health related quality of life (HRQoL) is a multidimensional concept that includes global health perspectives, symptom status, functional status, biological and physical variables, individual and environmental characteristics and general health perception [10].

According to the World Health Organization (WHO), QoL is defined as an individuals’ perception of their position in life in the context of the culture and value systems in which they live and in relation to their goals, expectations, standards and concerns [9]. This definition considers individuals’ satisfaction on their physical, psychological, social relationships, environment, and spiritual aspects of their life [11].

Quality of life is affected by several clinical and socio-demographic factors. Some of the factors that predict QoL were baseline CD4 lymphocyte count [12, 13], time since HIV diagnosis [12], poor social support [14], depression [15–18], unemployment [19], older age [20] and being female [21].

Many studies have provided important information on the correlates of HRQoL during HIV infection. Many studies have documented significant improvements in QoL during ART [20, 22, 23]. There is however knowledge gap on the HRQoL of HIV-VL coinfection as a treatment outcome. Therefore, the objective of the present study was to assess the level of HRQoL and its predictors and/ or correlates among HIV infected adults with and without VL.

## Methods

### Study design

Facility based cross-sectional study design was employed to assess the level of HRQoL and its predictors and/or correlates among HIV infected adults with and without VL who visited the health facilities in Northwest Ethiopia.

### Study settings and population

From the VL treatment centers found in the Northwest Ethiopia, three hospitals and one health center were selected purposely considering the availability of invasive VL diagnostic methods such as demonstration of parasite from spleen/lymph node aspiration or positive serology test if the patient has no VL history. In addition to VL diagnostic method, we also considered the availability of Fluorescence Activate Cell Sorting (FACS) count machine for CD4 count and CBC (complete blood count) machine. Hospitals and Health centers found in the study area that fulfilled the above considerations were considered as clusters (units). Cluster sampling technique was employed in order to include HIV-VL coinfected study participants. Therefore, Abdrafi Health center, Metema Hospital, Humera Hospital, and University of Gondar Hospital were the four selected clusters. All HIV-VL coinfection diagnosed patients who visited these facilities were included in the study. The selected Hospitals and Health center are the only health facilities that have well organized VL diagnosis and treatment centers found in the study area. The excluded health facilities in our study have not yet started diagnosing and treating VL patients. If HIV-VL coinfected patients visited these health facilities then they will be referred to one of the selected health facilities.

The study was carried out at four different sites in Northwest Ethiopia. The first site was Abdrafi inpatient kala-azar treatment center located in Abdrafi; at this health center medical services are provided for patients with leishmaniasis, HIV-VL coinfection and snake bite. The second site was Kala-azar Treatment and Research Center in the University of Gondar Hospital located in Gondar; at this center both outpatient and inpatient medical services are provided for patients with leishmaniasis and HIV-VL coinfection in addition to the comprehensive medical service from other units of the University of Gondar Hospital. The third site was Kahsay Aberra Hospital located in Humera kala-azar treatment center; at this center both outpatient and inpatient medical services are provided for patients with leishmaniasis, HIV-VL coinfection and many other hospital level services. The fourth site was Metema Hospital located in Metema kala-azar treatment center; at this center both outpatient and inpatient medical services are provided for patients with leishmaniasis, HIV-VL coinfection and many other hospital level services.

Sample size for the two groups was determined using WINPEPI (Window program for Epidemiologist) [24]. In a recent study, the mean score of general QoL among HIV infected patients who were taking highly active antiretroviral therapy in Jimma hospital was 87 [25]. Due to absence of data, we assumed HIV-VL coinfected patients would have a 5% lower mean score of general health as compared to HIV patients. With a power of 80%, 95% CI, a 1:3 ratio of HIV-VL coinfected patients versus HIV patients, and a 10% for non-response rate, the sample size was 620 (155 coinfected patients and 465 HIV patients).

The study populations were all HIV-VL coinfected and HIV infected patients who were attending their treatment in the treatment facilities found in the Northwest Ethiopia. The study period was from October, 2015 to September, 2016. During the study period, all new VL infected patients were identified and then they were screened for HIV infection. Only HIV-VL coinfected patients who were in the intensive phase of anti-VL treatment during the study period were included. For each HIV-VL coinfected patient, 3 HIV infected patients without VL were selected using simple random sampling technique. Participants who were mentally incompetent, age < 15 years, the presence of opportunistic infections or a known chronic illness like diabetic mellitus and hypertension were excluded from the study.

### Measurements

Diagnosis of VL was conducted according to the guidelines for the diagnosis of leishmaniasis in Ethiopia [26]. The WHO case definition of VL was used as a starting point; history of fever for > 2 weeks, malaria excluded, in combination with wasting and either splenomegaly or lymphadenophaty [27]. A patient whose illness met this case definition and who had no previous VL treatment was diagnosed serologically by positive rK39 rapid diagnostic test (Diamed-IT-Leish, DiaMed AG) [28]. Patients with previous VL history underwent splenic or lymph node aspiration and VL confirmed parasitologicaly. A severely ill patient with a negative rK39 test was aspirated without delay, So that a diagnosis could be made as quickly as possible.

Provider-initiated testing and counseling for HIV was offered to all VL patients. The HIV diagnosis was based on the national algorithm with two serial positive rapid test results; The KHB (Shanghai Kehua Bio-engineering Ltd, 2008, Shanghai, China) HIV test was used to diagnose HIV. For positive results, confirmation were done using STAT-PAK test (chembio diagnostic system Inc, 2008, New York, USA). In case of discrepancy between the two tests, Uni-Gold™ (Trinity Biotech PLC, Bray, Ireland) was used as a tie breaker. As VL is considered a stage IV-defining illness in HIV patients [27, 29], all patients were given ART as soon as they were stabilized from their acute illnesses. ART regimens follow the national guidelines: tenofovir-lamivudine-efavirenz; zidovudine-lamivudine-efavirenz; or zidovudine-lamivudine-nevirapine [30]. Second-line ART consists of protease inhibitor-based combination regimens.

QoL was measured at baseline through face to face interviews using the short amharic version of the World Health Organization QoL Instrument for HIV infected patients (WHOQoL HIV-Brief) [31]. This QoL instrument has been described in TB/HIV coinfection and its impact on quality of life article [16]. In brief, it consisted of 31 Likert scale questions in 6 domains of QoL: physical health (4 items); psychological wellbeing (5 items); social relationship (4 items); environmental health (8 items); level of independence (4 items) and spiritual health (4 items). There were two questions about general QoL and perceived general health.

Common Mental Disorder (CMD) was measured using the Kessler 10 scales [32]. This instrument has 10 questions each asking the respondent how often they experienced symptoms during the previous 30 days and containing 5-point Likert scales (1 = never, 2 = a small part of the time, 3 = some of the time, 4 = most of the times, 5 = all of the time). The Kessler-10 scale was validated in Ethiopia and used extensively [33].

Data on demographic factors, clinical and treatment related factors were collected by using structured and pre-tested questionnaire which was developed by the investigators. The structured questionnaire was prepared in English version and translated into Amharic (local language) and again back to English to confirm the correctness of the translation and for analysis purpose. The data collectors were 4 nurses and we also recruited 4 physicians as supervisors for the data collectors.

### Data analysis

Each completed questionnaire was checked visually for completeness before fed to computer. The data was entered into SPSS version 21, data clean up and cross-checking was done and it was analyzed by using SPSS version 20. Domain scores in the WHOQoL-HIV-Brief were scaled in positive direction with higher score denoting good quality of life. Negative questions like pain and discomfort were recorded so that higher scores reflect better QoL. Mean scores of items within each domain was used to calculate the domain score. Mean scores were then multiplied by 4 to make domain scores comparable with the scores used in the World Health Organization QoL (WHOQoL-100). We used *T*-test and F-test to compare means between groups.

Independent variables which were associated with each of the six HRQoL domains were first identified using bivariate linear regression analysis. Then all the variables that yielded *p*-values of <0.2 were fitted in the multivariate linear regression model. We checked for violations of regression model assumptions by inspection of plots of residuals versus predicted values, outliers and multicollinearity. Results of the regression analysis are expressed in un-standardized coefficient (beta). Beta coefficients are measured in units of standard deviation and refer to the average change in the dependent variable for a unit increase in the predictor variable.

## Results

### Characteristics of the study participants

A total of 590 study participants were included in the study with response rate of 95%. Of the 590 patients included in our study 125 (21%) were HIV-VL coinfected. Thirty participants refused to participate in the study. The mean age (±SD) was 34.3 (±7.4) year for HIV-VL coinfected and 36.4 (±8.8) year for HIV infected study participants.

Males and urban residents were more likely to be infected by visceral leishmaniasis (VL) than their counter parts (*p* = 0.001). HIV-VL coinfected patients were more likely to have lower CD4 lymphocyte and observed hemoglobin (Hb) level than HIV patients (*p* = 0.001). All the coinfected patients were WHO stage IV and 65% of HIV infected patients were stage I. All HIV patients and 51% of the coinfected patients were taking ART during the study period (Table 1).

**Table 1:** Socio-demographic and clinical characteristics of the study population in Northwest Ethiopia

Variables	HIV-VL coinfected patients (*N* = 125) Number (%)	HIV infected patients (*N* = 465) Number (%)	*P*-value
Age in Years			0.001
15–24	1 (0.8%)	22 (4.7%)	
25–34	72 (57.6%)	177 (38.1%)	
≥ 35	52 (41.6%)	266 (57.2%)	
Sex			0.001
Male	121 (96.8%)	178 (38.3%)	
Female	4 (3.2%)	287 (61.7%)	
Residence			0.001
Urban	85 (68.0%)	438 (94.2%)	
Rural	40 (32.0%)	27 (5.8%)	
Educational Status			0.001
Uneducated	65.6 (65.6%)	210 (45.2%)	
Primary cycle	23 (18.4%)	92 (19.8%)	
Secondary and above	20 (16.0%)	163 (35.1%)	
Occupation			0.001
Farmer	38 (30.4%)	26 (5.6%)	
Daily Labor	65 (52.0%)	74 (15.9%)	
Employed	16 (12.8%)	131 (28.2%)	
No Job	1 (0.8%)	63 (13.5%)	
Merchant	2 (1.6%)	52 (11.2%)	
Housewives	3 (2.4%)	119 (25.6%)	
Religion			0.481
Christian	112 (89.6%)	426 (91.6%)	
Muslim	13 (10.4%)	39 (8.4%)	
Ethnicity			0.001
Amhara	82 (65.6%)	404 (86.9%)	
Tigray	38 (30.4%)	48 (10.3%)	
Others	5 (4.0%)	13 (2.8%)	
Marital status			0.001
Single	52 (41.6%)	68 (14.6%)	
Married	40 (32.0%)	234 (50.3%)	
Separated	10 (8.0%)	44 (9.5%)	
Divorced	19 (15.2%)	64 (13.8%)	
Widowed	4 (3.2%)	55 (11.8%)	
WHO staging			0.001
Stage I	-	302 (64.9%)	
Stage II	-	76 (16.3%)	
Stage III	-	78 (16.8%)	
Stage IV	125 (100%)	9 (1.9%)	
On Antiretroviral therapy			0.001
Yes	64 (51.2%)	465 (100%)	
No	61 (48.8%)		
CD4 count (mg/dl)			
Median (Range) = 340 (from 30 to 1652)			0.001
≤ 100	71 (57.7%)	20 (4.3%)	
101–200	38 (30.9%)	47 (10.1%)	
≥ 200	14 (11.4%)	398 (85.6%)	
Observed Hb level (mg/dl)			
Mean (±SD) =12.8 (±2.8)			0.001
< 12.8	120 (96.8)	101 (23.1%)	
≥ 12.8	4 (3.2)	336 (76.9%)	
Spleen size (cm)			
< 15	109 (94.8%)	NA	
≥ 15	6 (5.2%)	NA	

### Internal consistency of the WHOQOL-HIV

To measure internal consistency, the Cronbach’s alpha was calculated for each domain of the instrument. Most domains of the amharic version of the WHOQoL-HIV had a high value of Cronbach’s alpha (α > 0.7). However, spiritual health had a lower internal consistency (α = 0.64) as compared to others (Table 2).

**Table 2:** Internal consistency of the Amharic version of the WHOQOL-HIV questionnaire

Domain	Coefficient for internal consistency (Cronbach’s alpha)
Physical	0.82
Psychological	0.88
Social relation	0.73
Environmental	0.88
Level of independence	0.77
Spiritual	0.64

### The Kessler scale

The correlation between items in the Kessler scale ranged from 0.36 to 0.68 with no multicollinearity and redundancy. The internal consistency of the Kessler scale was high (Cronbach’s α = 0.88).

Inter domain correlations showed that there were statistically significant associations between domains. However, there was no correlation between psychological health, social relation, and environmental health with spiritual health. There was a week correlation between spiritual health and level of independence (Table 3).

**Table 3:** Correlations between the domains of the Amharic version of the WHOQOL-HIV questionnaire

Domains	PH	Psy	Soc	Env	Ind	Spir
PH	1					
Psy	0.374^a^	1				
Soc	0.303^a^	0.676^a^	1			
Env	0.344^a^	0.728^a^	0.661^a^	1		
Ind	0.570^a^	0.594^a^	0.556^a^	0.586^a^	1	
Spir	0.348^a^	0.056	0.053	0.093^b^	0.243^a^	1

*PH* Physical health, *Psy* Psychological health, *Soc* Social relation, *Env* Environment, *Ind* Level of Independence, *Spir* Spiritual

^a^Correlation is significant at the 0.01 level (2-tailed), ^b^Correlation is significant at the 0.05 level (2-tailed)

We found correlations between the WHOQoL domains and the Kessler scale. Strong correlations was observed between physical health (correlation coefficient or *r* = −0.633, *p* = 0.001), level of independence (*r* = −0.509, p = 0.001) and spiritual health (*r* = −0.403, *p* = 0.001) with Kessler scale. Psychological health, social relation, and environmental domains had a correlation coefficient of −0.335, −0.295 and −0.350 with Kessler scale respectively (*p*-value = 0.001).

### Quality of Life

HIV-VL coinfected patients had a lower mean score in all domains indicating poor quality of life. Mean scores for physical health, social relationship and environmental health among coinfected patients were 10.42, 9.71 and 9.66 respectively. The mean (SD) depressive-symptoms scale score were higher 2.67 (±0.7) for HIV-VL coinfected patients than HIV patients1.61 (±0.5) (*p* = 0.001) (Table 4).

**Table 4:** Comparison of Quality of life of HIV infected patients with and without Visceral Leishmaniasis in Northwest Ethiopia

Quality of life Domain	HIV-VL coinfection (*n* = 125) Mean (±SD)	HIV infected patients (*n* = 465) Mean (±SD)	*P*-value
Physical Health	10.42 (±3.5)	17.43 (±2.8)	0.001
Psychological Health	10.68 (±3.6)	12.60 (±3.4)	0.001
Social Health	9.71 (±3.1)	11.44 (±3.4)	0.001
Environmental Health	9.66 (±3.1)	11.53 (±2.9)	0.001
Level of independence	9.46 (±3.2)	13.34 (±2.8)	0.001
Spiritual Health	11.49 (±3.3)	15.06 (±3.4)	0.001
Depressive-symptoms	2.67 (±0.7)	1.61 (±0.5)	0.001

### Predictors of QOL

Tables 5 and 6 show the results of the bivariate and multivariate linear regression analysis for predictors of the HRQoL domains respectively. The final multivariate regression model did not suggest multicollinearity problems.

**Table 5:** Bivariate linear regression analysis of predictors and/or correlates of HRQoL of HIV infected patients with and without VL in Northwest Ethiopia

Patient characteristics	HRQoL domains											
	Physical domain (β-coefficient)		Psychological domain (β-coefficient)		Social domain (β-coefficient)		Spiritual domain (β-coefficient)		Level of independence (β-coefficient)		Environmental domain (β-coefficient)	
	HIV-VL (*n* = 125)	HIV (*n* = 465)	HIV-VL (*n* = 125)	HIV (*n* = 465)	HIV-VL (*n* = 125)	HIV (*n* = 465)	HIV-VL (*n* = 125)	HIV (*n* = 465)	HIV-VL (*n* = 125)	HIV (*n* = 465)	HIV-VL (*n* = 125)	HIV (*n* = 465)
Age	−0.096**	−0.022*	−0.035	−0.005	−0.011	0.001	0.037	0.019	−0.035	−0.012	0.013	0.027*
Sex												
Male	0.435	−0.097	0.291	−0.314	−0.298	0.269	1.021	−0.183	1.512	−0.119	0.229	0.153
Female	Ref.	Ref.	Ref.	Ref.	Ref.	Ref.	Ref.	Ref.	Ref.	Ref.	Ref.	Ref.
Education												
Not educated	Ref.	Ref.	Ref.	Ref.	Ref.	Ref.	Ref.	Ref.	Ref.	Ref.	Ref.	Ref.
Educated	1.225*	0.745***	0.535	1.830***	0.971*	1.551***	0.036	−0.138	0.108	0.998***	−0.037	1.738***
Marital status												
Married	−0.620	1.031***	0.056	0.464*	0.313	1.573***	0.018	0.029	−0.241	0.680**	0.163	0.565**
Not married	Ref.	Ref.	Ref.	Ref.	Ref.	Ref.	Ref.	Ref.	Ref.	Ref.	Ref.	Ref.
Residence												
Urban	0.669	0.837*	0.502	0.519	1.121*	1.058*	−0.606	0.892*	0.057	0.791*	0.866*	0.306
Rural	Ref.	Ref.	Ref.	Ref.	Ref.	Ref.	Ref.	Ref.	Ref.	Ref.	Ref.	Ref.
CD4 count												
<201	Ref.	Ref.	Ref.	Ref.	Ref.	Ref.	Ref.	Ref.	Ref.	Ref.	Ref.	Ref.
> = 201	1.671*	0.602*	2.643***	1.114*	1.531*	0.498	0.255	0.404	2.775***	0.918**	2.349***	0.641
Hb level												
<12.8	Ref.	Ref.	Ref.	Ref.	Ref.	Ref.	Ref.	Ref.	Ref.	Ref.	Ref.	Ref.
> = 12.8	−0.091	0.836***	−1.737	0.737**	−0.736	0.750**	−0.504	0.022	−2.804*	0.221	−1.461	0.861***
Duration of ART	0.332***	0.179***	0.501***	0.287***	0.342***	0.231***	0.407***	−0.043	0.454***	0.121***	0.306***	0.262***
Kessler depressive symptoms	−2.351***	−2.641***	−3.061***	−1.560***	−2.205***	−0.969***	−2.199***	−1.460***	−2.209***	−1.897***	−2.447***	−1.256***

*Ref.* reference category

*p-value < 0.20; **p-value < 0.05; ***p-value <0.01

**Table 6:** Multivariate linear regression analysis of predictors and/or correlates of HRQoL of HIV infected patients with and without VL in Northwest Ethiopia

Patient characteristics	HRQoL domains											
	Physical domain (β-coefficient)		Psychological domain (β-coefficient)		Social Domain (β-coefficient)		Spiritual domain (β-coefficient)		Level of independence (β-coefficient)		Environmental domain (β-coefficient)	
	HIV-VL (*n* = 125)	HIV (*n* = 465)	HIV-VL (*n* = 125)	HIV (*n* = 465)	HIV-VL (*n* = 125)	HIV (*n* = 465)	HIV-VL (*n* = 125)	HIV (*n* = 465)	HIV-VL (*n* = 125)	HIV (*n* = 465)	HIV-VL (*n* = 125)	HIV (*n* = 465)
Age	−0.083*	−0.031*										
Sex												
Male												
Female												
Education												
Not educated				Ref.		Ref.				Ref.		Ref.
Educated				1.432**		1.142**				0.589*		1.378**
Marital status												
Married		0.816**				1.415**				0.495*		
Not married		Ref.				Ref.				Ref.		
Residence												
Urban												
Rural												
CD4 count												
< 201				Ref.						Ref.		
> =201				1.034*						0.843*		
Hb level												
< 12.8												
> =12.8												
Duration of ART		0.144**	0.224*	0.232**		0.184**	0.221*		0.258*			0.197**
Kessler depressive symptoms	−2.118**	−2.489**	−2.670**	−1.220**	−1.981**	−0.603*	−1.887**	−1.449**	−1.630**	−1.724**	−2.275**	−0.925**
*p*-value of the final multivariate regression model	*P* < 0.01	*P* < 0.01	*P* < 0.01	*P* < 0.01	*P* < 0.01	*P* < 0.01	*P* < 0.01	*P* < 0.01	*P* < 0.01	*P* < 0.01	*P* < 0.01	*P* < 0.01
Adjusted-R^2^	0.239	0.288	0.355	0.148	0.261	0.125	0.232	0.047	0.278	0.150	0.302	0.155

*Ref.* reference category

*p – value < 0.05; **p – value < 0.01

In the bivariate analysis duration of ART and Kessler depressive-symptoms were associated with HRQoL across all the domains for both groups. Observed Hb level ≥12.8 also strongly associated with physical, psychological, social and environmental domain for HIV infected group. As clearly indicated in Table 5, the variables with *p*-value <0.2 were entered into the multivariate analysis of each HRQoL domains.

A higher level of depressive-symptoms was strongly and consistently associated with a lower HRQoL across all the domains of both groups. Also, in HIV infected patients a longer duration in ART was associated with higher HRQoL domains except for the spiritual and level of independence domains. With regard to HIV-VL coinfected patients, a longer duration in ART was associated with psychological, spiritual and level of independence domains of HRQoL.

Having higher CD4 count (≥201 mg/dl) was associated with higher HRQoL on psychological and level of independence domains of HIV infected patients. Higher CD4 count has no association in all the domains of HRQoL of HIV-VL coinfected patients.

Being educated in HIV infected patients was associated with higher HRQoL on psychological, social, level of independence and environmental domains. There was no association found between education and HRQoL domains for HIV-VL coinfected patients.

Most demographics, clinical and treatment related variables were not associated with HRQoL domains or associated with fewer HRQoL domains for HIV infected patients. In HIV-VL coinfected patients, no statistically significant associations were found between HRQoL and employment, sex, education, marital status, residence, CD4-cell count and Hb level.

## Discussion

In this study we compared the HRQoL of persons with HIV infection with and without VL. Coinfected patients had a lower quality of life in all the domains of the WHOQoL-HIV as compared to people living with HIV without VL. The concurrent occurrence of these two diseases in a person can decrease the quality of life by affecting the physical, social and mental wellbeing. The reason for this is VL accelerates HIV replication and disease progression, mainly by chronic immune stimulation [3]. In other studies, it was reported that HIV patients when coinfected with other disease such as Tuberculosis (TB) had a lower quality of life as compared to HIV infected patients without TB coinfection [16].

It is difficult to compare the levels of HRQoL of HIV-VL coinfected patients in this study with HRQoL of HIV-TB coinfected patients since the nature and characteristics of these two diseases (TB and VL) are entirely different. Though, compared to the study conducted among TB and HIV coinfected patients in East and Southwest Ethiopia [16], HIV-VL coinfected patients in our study reported lower levels of HRQoL in all domains of HIV-TB coinfected patients. HIV infected patients without VL in our study also reported lower HRQoL in all domains except for the physical health domain as compared to HIV infected patients without TB. This is not meant strict comparison, however, as patients in this study coinfected with VL and there may also be additional differences of perception of personal beliefs. Nevertheless, our study result of HIV infected patients without VL is comparable with findings from HRQoL of HIV infected adults receiving cART in Addis Ababa [17] and the baseline finding of prospective longitudinal study done in Northwest Ethiopia [34].

A higher level of depressive-symptoms was most strongly and consistently associated with a lower HRQoL across all the domains, both in-terms of the magnitude of relationship and in the number of HRQoL domains associated with it in both HIV-VL coinfected and HIV infected without VL patients. This finding is in line with previous studies conducted in variety of research settings [15, 17, 35]. Therefore, this study gives additional evidence in order to design intervention to alleviate depressive-symptoms in HIV and HIV-VL infected patients.

A longer duration of cART for HIV infected patients was independently associated with better HRQoL domains, except with the spiritual and level of independence domains. A longer duration of cART for HIV-VL coinfected patients was associated with psychological, spiritual and level of independence domains of HRQoL. In line with this finding, there was a study conducted by Casado et al. [36] assessed longitudinal changes in QOL for three months after beginning cART. There is also another study conducted by Manneiner et al., [20], who reported significant improvement in QoL after 1 to 4 months of treatment with cART, and this improvement persisted at 12 months. On the other hand, Wouters et al. [37] reported that additional cART did not further improve the QoL of patients who had received cART for less than 6 months at baseline.

Among the socio-demographic variables, older age was a significant predictor for poor physical health domain in both HIV-VL and HIV infected patients. This finding is in line with previous study results [17]. This might be because of physical functioning deteriorates as a result of aging or age related co-morbidities [38]. Being married HIV infected patients without VL was associated with higher HRQoL for physical, social and level of independence domains. There is a study [39] which have consistent finding with our study. It is believed that the physical, emotional and social support received from their partners likely led to improve QoL. There is evidence that showed support from outside the family cannot replace for what is missing in the family [39]. In contrast, there is also evidence on marital status had no significant association with any of the domains of HRQoL [40].

Educated HIV infected patients as compared to not educated was strongly associated with higher HRQoL on psychological, social, level of independence and environmental domains. This finding is in line with other country study among patients with HIV, the less educated had a lower quality of life [41]. There was no association found between education and all the domains of HRQoL for HIV-VL coinfected patients. Possibly, this might be explained by the small sample size or lower percentage of (i.e., 21%) of patients with evidence of VL at or after start of cART.

Having higher CD4 count (≥201 mg/dl) was associated with higher HRQoL on psychological and level of independence domains of HIV infected patients. This finding is in line with the previous study [13]. In contrast to these findings, we didn’t find a significant relationship between most of the domains of HRQoL of HIV infected patients and all the domains of HRQoL of HIV-VL coinfected patients and CD4-cell count. This finding is also in line with the previous studies done in Ethiopia [16, 17].

In contrast with other studies, we couldn’t find an association between WHO staging, employment, social support, source of income and other socio-demographic factors [16, 17, 41].

The findings of this study should be interpreted with some limitation. Being a cross-sectional study, causal inference cannot be made between HRQoL and independent variables especially the relationship between HRQoL and depression is complex and bidirectional. VL might induce depression but we didn’t include group of patients with VL alone to complement the relationship. Administering the questionnaire through face to face interview may have resulted socially desirable response. Therefore, social desirable bias might be introduced.

## Conclusion

HIV-VL coinfected patients had a poor quality of life in all the domains of the WHOQOL-HIV instrument. Depression, duration of ART and education were strongly associated with the quality of life. The governmental and non-governmental organizations working in VL control programs should design strategies to improve the quality of life of HIV-VL coinfected patients. Depression should be targeted for intervention to improve the quality of life. To increase adherence of cART and quality of life, patients should be counseled and educated.

## Abbreviations

ART: Antiretroviral therapy
ARV: Antiretroviral drug
CBC: Complete blood count
CD4: Cluster of differentiation 4
FACS: Fluorescence Activate Cell Sorting
HRQoL: Health related quality of life
QoL: Quality of life
rK39: Recombinant K 39
SPSS: Statistical package for social science
VL: Visceral Leishmaniasis
WHO: World Health Organization
WHOQOL-HIV: World Health Organization Quality of Life – Human Immunodeficiency Virus
WINPEPI: Window program for Epidemiologist
